# CB_1_ Allosteric Modulator Org27569 Is an Antagonist/Inverse Agonist of ERK1/2 Signaling

**DOI:** 10.1089/can.2016.0028

**Published:** 2016-12-01

**Authors:** Thomas F. Gamage, Johnathon C. Anderson, Mary E. Abood

**Affiliations:** ^1^ Center for Substance Abuse Research, Lewis Katz School of Medicine, Temple University, Philadelphia, Pennsylvania.; ^2^ Department of Anatomy and Cell Biology, Lewis Katz School of Medicine, Temple University, Philadelphia, Pennsylvania.

**Keywords:** allosteric, cannabinoid, CB_1_, ERK, signaling, Org27569

## Abstract

**Introduction:** Allosteric modulation of cannabinoid type-1 receptors (CB_1_) is a novel means through which signaling bias may be exerted. Org27569 remains the most-characterized CB_1_ allosteric modulator, yet there are conflicting reports regarding its effects on extracellular signal-regulated kinase (ERK) signaling. We conducted a systematic evaluation of Org27569's effects on cannabinoid agonists and ERK signaling.

**Materials and Methods:** HEK293 cells transfected with the human cannabinoid type-1 receptor (hCB1) were treated with Org27569 alone or in combination with the endocannabinoid 2-arachidonoylglycerol (2-AG), the synthetic cannabinoid CP55,940, or the phytocannabinoid delta-9-tetrahydrocannabinol (THC) and ERK activation was measured by western blot. Overnight treatment with pertussis toxin (PTX) was used to determine the role of G_i/o_ in Org27569's inverse agonist effects. HEK293 cells transfected with green fluorescent protein tagged rat CB_1_ receptor were used to assess effects of Org27569 on CP55,940-induced receptor internalization. Subcellular fractionation was used to determine effects of Org27569 on ERK phosphorylation in both nuclear and cytosolic compartments.

**Results:** We found that Org27569 is an antagonist of hCB_1_-mediated ERK signaling in HEK293 cells where it fully blocks CP55,940-but does not completely inhibit THC- and 2-AG-stimulated ERK1/2 activation following 5 min treatment. In rat CB_1_ HEK293 cells, CP55,940 (1 μM) treatment produced a significant increase in puncta at 20, 40, 60, and 120 min, consistent with receptor internalization. Org27569 (10 μM) co-treatment prevented internalization at each time point and alone had no effect. Org27569 reduced basal ERK phosphorylation in hCB_1_ HEK293 cells but not in untransfected cells following 20 min treatment. Overnight treatment with PTX abated this response. Following subcellular fractionation, Org27569 produced a significant decrease in ERK phosphorylation in the nuclear-enriched and cytosolic fractions.

**Conclusions:** These data are consistent with previous studies demonstrating that CB_1_-mediated ERK1/2 activation is G_i/o_-dependent and that Org27569 is an inverse agonist of CB_1_ receptors. Abrogation of Org27569's ability to reduce basal ERK phosphorylation following treatment with PTX and lack of inverse agonist effects in untransfected HEK293 cells demonstrates that Org27569 acts via CB_1_-G_i/o_ to produce this effect. To our knowledge, this is the first reported demonstration of inverse agonism of ERK signaling by Org27569.

## Introduction

The endocannabinoid system currently comprises two G-protein-coupled receptors (GPCR), cannabinoid type-1 (CB_1_)^1^ and type-2 (CB_2_),^2^ endogenous ligands (endocannabinoids), including *N*-arachidonoylethanolamine (anandamide; AEA)^3^ and 2-arachidonoylglycerol (2-AG),^4^ and the regulatory enzymes for the synthesis and degradation of endocannabinoids.^5^

While the phytocannabinoid delta-9-tetrahydrocannabinol (THC), the primary psychoactive constituent of cannabis,^6^ acts on the CB_1_ receptor to exert its abuse-related effects, the therapeutic effects of cannabinoids are also mediated, in part, by this receptor,^5^ and therefore, it is of much interest for the development of pharmacotherapeutics. The CB_1_ receptor couples to G_i/o_-proteins, which on activation lead to (1) inhibition of adenylyl cyclase and L-, N-, and P/Q-type voltage-gated calcium channels^7–9^ and (2) activation of inwardly rectifying potassium channels^10^ and (3) mitogen-activated protein kinase/extracellular signal-regulated kinase (MAPK/ERK).^11^

The pleiotropic nature of GPCR signaling^12^ has led to the pursuit of compounds that can selectively activate specific signaling pathways to better study their contribution to the effects of cannabinoids and to develop pharmacotherapeutic strategies, which are efficacious yet lack the concomitant adverse effects typically observed with CB_1_ agonism. Classically, CB_1_ receptor signaling has been focused on using compounds that compete for an orthosteric binding site, thus offering a limited approach, in which a single compound activates a set response depending on its own intrinsic signaling biases. Agonists of the CB_1_ receptor exhibit bias/functional selectivity, in which certain signaling pathways can be preferentially activated.^13^

Allosteric modulation offers an additional layer of control over receptor signaling, in that binding to an allosteric site can further alter receptor conformation to affect agonist binding/efficacy and signaling specificity or impart signaling on its own in the absence of an agonist. Allosteric modulation of the CB_1_ receptor is a recent development in cannabinoid pharmacology with functionally negative^14,15^ and positive^16–19^ allosteric modulators having been reported only within the last decade.

The most characterized compound to date, Org27569, has been reported to act as an insurmountable antagonist/inverse agonist of CP55,940-stimulated [^35^S]GTPγS binding,^14,20,21^ while exhibiting positive binding cooperativity with [^3^H]CP55,940.^14^ Org27569 also attenuates cannabinoid agonists' ability to inhibit forskolin-stimulated cAMP production^13,22^ and acts as a CB_1_ inverse agonist, increasing cAMP levels over forskolin stimulation in a pertussis toxin (PTX)-sensitive manner.^22^ The effects of Org27569 on ERK1/2 signaling are unclear as Org27569 has been reported to act as either an allosteric agonist of ERK1/2 signaling via beta-arrestin1,^23^ or G_i/o_,^20^ or as an allosteric antagonist.^13^

CB_1_ activation of ERK1/2 occurs through a number of mechanisms^24^ in a time-dependent manner, with peak effects occurring at ∼5 min when examined in CB_1_ HEK293 cells.^25^ Following activation, ERK1/2 translocates to the nucleus where it regulates gene expression,^26^ which impacts a number of functions, including those important for synaptic plasticity^27^ and the development of cannabinoid tolerance.^28^ Because there are separate pools of ERK1/2—cytoplasmic and nuclear—it is possible that measurement of ERK phosphorylation in total cell lysates could obscure differences in phosphorylation states that exist between these two compartments.

In this study, we therefore examined the effects of Org27569 alone on ERK1/2 phosphorylation in both cytoplasmic and nuclear compartments to ensure that changes in phosphorylation states in one compartment were not obscured by the other. ERK1/2 phosphorylation following Org27569 treatment was measured at 20 min to ensure we could observe a potential decrease, based on previous literature with other inverse agonists.^29,30^

To further investigate the aforementioned disparate findings of Org27569 effects on ERK signaling, we hypothesized that Org27569 would act as an antagonist/inverse agonist of ERK1/2 signaling, since previous literature indicates that Org27569 is a CB_1_ inverse agonist of [^35^S]GTPγS binding. Furthermore, since CB_1_-mediated ERK1/2 activation is G_i/o_ dependent^11,31^ and the CB_1_ antagonist/inverse agonist SR141716A elicits reductions in basal ERK1/2 phosphorylation^29^ that are PTX sensitive,^30^ we tested the PTX sensitivity of the ERK response elicited by Org27659.

## Materials and Methods

### Materials and reagents

CP55,940, delta-9-tetrahydrocannabinol (THC), SR141716A, 2-arachidonoylglycerol (2-AG), and Org27569 were provided by the National Institute on Drug Abuse (Bethesda, MD) and were dissolved in DMSO and diluted to a final working concentration of 0.1–0.2%. PTX (Calbiochem, San Diego, CA) was dissolved in MilliQ filtered water.

### Cell cultures and transfection

Vendor authenticated human embryonic kidney (HEK293; American Type Culture Collection, Rockville, MD) cells were cultured in 5% fetal bovine serum (FBS) defined (Hyclone Labs, Logan, UT) in Dulbecco's modified Eagle's medium (DMEM; Corning Cellgro, Manassas, VA) at 37°C and 5% CO_2_. Cell lines were generated as previously described^32^ by transfection of the untagged hCB_1_ or N-terminally green fluorescent protein (GFP)-tagged rat CB_1_ receptor with Lipofectamine 2000 (Life Technologies, Gaithersburg, MD). Cells were maintained in a culture medium with geneticin (0.5 mg/mL; G418) and not used after 25 passages.

### Cell treatment and phospho-ERK quantification

Cells were plated in poly-d-lysine (PDL)-coated 6-well plates or 100-mm dishes (for fractionation experiments) to a confluency of ∼70–80% and serum starved for 24 h before all treatments. For internalization experiments, cells were plated on PDL-coated cover-slips in a 24-well format. For experiments examining G_i/o_ protein mediation, cells were exposed to PTX (200 ng/mL) for 18 h before drug treatment when appropriate.

Cells were treated by application of vehicle or antagonist followed immediately by application of vehicle or agonist and incubated in serum-free DMEM at 37°C for times indicated, then washed once with ice-cold phosphate-buffered saline (PBS), and lysed with buffer containing HEPES (50 mM), NaCl (150 mM), EDTA (1 mM), EGTA (1 mM), glycerol (10%), Triton X-100 (1%), MgCl_2_ (10 μM), NaF (25 mM), Na_3_VO_4_ (1 mM), para-nitrophenyl phosphate (20 mM) and EDTA-free protease inhibitor. Lysates were incubated on ice for 30 min, then centrifuged at 16,000 *g* for 30 min at 4°C, and supernatants were collected.

Protein amount (20 μg) was determined by the Bradford method, separated by 10% SDS-PAGE, and transferred to nitrocellulose membranes. Membranes were blocked for 1 h with the Odyssey blocking buffer (1:1, PBS:Odyssey blocking buffer) and incubated at room temperature for 2 h or overnight at 4°C in primary antibodies directed at phospho-ERK1/2 (Sigma Aldrich M8159; 1:5000) and total ERK1/2 (Sigma Aldrich M5670; 1:8000).

Membranes were rinsed thrice in 0.1% Tween-20 in PBS before incubating with LiCor secondary antibodies (IRDye^®^ 800CW Goat anti-Mouse; 1:6666; IRDye 680RD Goat anti-Rabbit; 1:10,000) at room temperature for 1 h. Blots were imaged using LiCor Odyssey imaging software, and phospho-ERK signal was normalized to total ERK and data expressed as percent of vehicle control. All data points are means of a minimum of three separate experiments run in duplicate or triplicate.

### Receptor internalization

For internalization experiments, GFP-rat CB_1_ HEK293 cells were plated on PDL-coated cover-slips in the 24-well format and treated with vehicle (0.2% DMSO), CP55,940 (1 μM), Org27569 (10 μM), or both for 20–120 min. Cell images were taken blinded, converted to grayscale in Photoshop CS5, and then analyzed in ImageJ. Background was subtracted by using a “rolling ball” algorithm (rolling=3). Puncta within the range (size=3–400, circularity=0.01–1.00) were counted. These parameters were used based on image resolution and brightness. The same conditions were applied to all the images and image analyses.

### Subcellular fractionation

Cells were grown and treated as described above on 100-mm dishes and washed once with ice-cold PBS. Cells were then lysed by addition of 300–500 μL of fractionation buffer (250 mM sucrose, 20 mM HEPES, 10 mM KCl, 1.5 mM MgCl_2_, 1 mM EDTA, 1 mM EGTA, 1 mM DTT, phosphatase and protease inhibitors). Lysate was passed through a 26-gauge syringe 10 times and then incubated on ice for 30 min. The nuclear-enriched fraction was pelleted following centrifugation at 700*g* for 5 min and rinsed twice in fractionation buffer then centrifuged again and reconstituted in nuclear buffer (fractionation buffer containing 10% glycerol and 0.1% SDS) and sonicated at a setting of two-continuous.

The initial S1 fraction was centrifuged at 10,000 *g* for 10 min and the resulting S2 fraction served as the cytosolic/membrane fraction. Protein was determined by the NanoDrop™ A280 assay (Thermo Scientific, Wilmington, DE). Nuclear fraction enrichment was established by immunoblotting for lamin A/C (1:2000; Cell Signaling Technologies).

### Data analysis

Data were normalized as percent vehicle control and analyzed by one- or two-way ANOVA with Newman–Keuls and Bonferroni *post hoc* tests, and data were considered significant with *p*<0.05. For concentration–response studies, curve fitting was done using GraphPad Prism 5.0 (GraphPad Software, Inc., San Diego, CA) nonlinear regression [log(inhibitor) vs. response]. Data reported are the mean±SEM of at least three independent experiments.

## Results

### Org27569 and SR141716A blocked CP55,940-induced ERK activation and receptor internalization

**FIG. 1.**
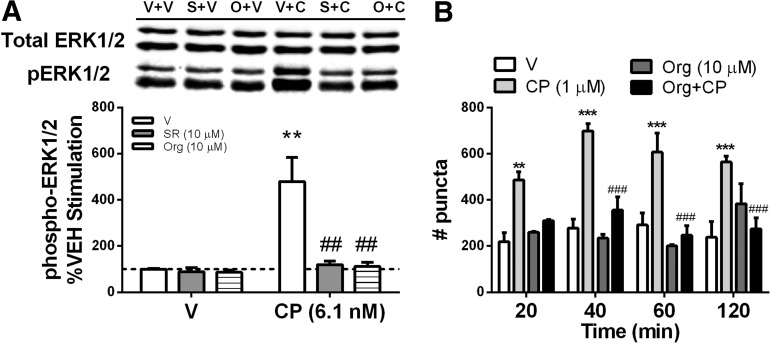
Org27569 and SR141716A blocked CP55,940-induced ERK activation and receptor internalization. **(A)** HEK293 cells stably transfected with the hCB_1_ receptor were exposed to the CB_1_ antagonist/inverse agonist SR141716A (10.0 μM) or the CB_1_ allosteric modulator Org27569 (10.0 μM) alone or in combination with CP55,940 (6.1 nM) for 5 min. **(B)** Org27569 prevented CP55,940-induced receptor internalization in HEK293 cells stably transfected with GFP-rat CB_1_ receptor. Protein was separated by SDS-PAGE and western blots for phospho-ERK normalized to total ERK. GFP-rat CB_1_ cells were seeded on PDL-coated cover-slips and treated with Org27569/CP55,940 or corresponding vehicle, imaged by a blinded observer, converted to grayscale and analyzed in ImageJ, which counted puncta. Data were analyzed by two-way ANOVA with Bonferroni *post hoc* tests. Data are the mean±SEM of at least three independent experiments. **p<0.01, ***p<0.001 compared to V+V control; ^##^*p*<0.01, ^###^*p*<0.001 compared to V+CP. CB_1_, cannabinoid type-1 receptor; CP, CP55,940; GFP, green fluorescent protein; hCB_1_, human CB_1_; ERK, extracellular signal-regulated kinase; PDL, poly-d-lysine; V, vehicle.

We first assessed the effects of Org27569 alone and in combination with the cannabinoid agonist CP55940 and included the selective CB_1_ antagonist SR141716A as a control (Fig. 1A). Following a 5-min incubation, Org27569 and SR141716A alone produced a small but nonsignificant reduction of ERK phosphorylation, whereas CP55,940 produced a robust increase in ERK phosphorylation (main effect: CP55,940 [*F*(1,12)=9.289, *p*<0.05]). However, both SR141716A and Org27569 prevented CP55,940-induced activation of ERK (main effect: [*F*(2,12)=6.593, *p*<0.05]; interaction effect: [*F*(2,12)=5.622, *p*<0.05]), suggesting that Org27569 can act as an antagonist of CB_1_-mediated ERK phosphorylation.

In addition, we sought to determine if Org27569 could prevent internalization of GFP-tagged rat CB_1_ receptors by the cannabinoid agonist CP55,940. We observed that CP55,940 (1 μM)-induced increases in puncta were prevented by Org27569 (10 μM) at every time point assessed (main effect: treatment [*F*(3,32)=44.8, *p*<0.0001]), suggesting that Org27569 prevented internalization of CB_1_ receptors (Fig. 1B).

### Org27569 antagonizes cannabinoid agonist-induced ERK activation

**FIG. 2.**
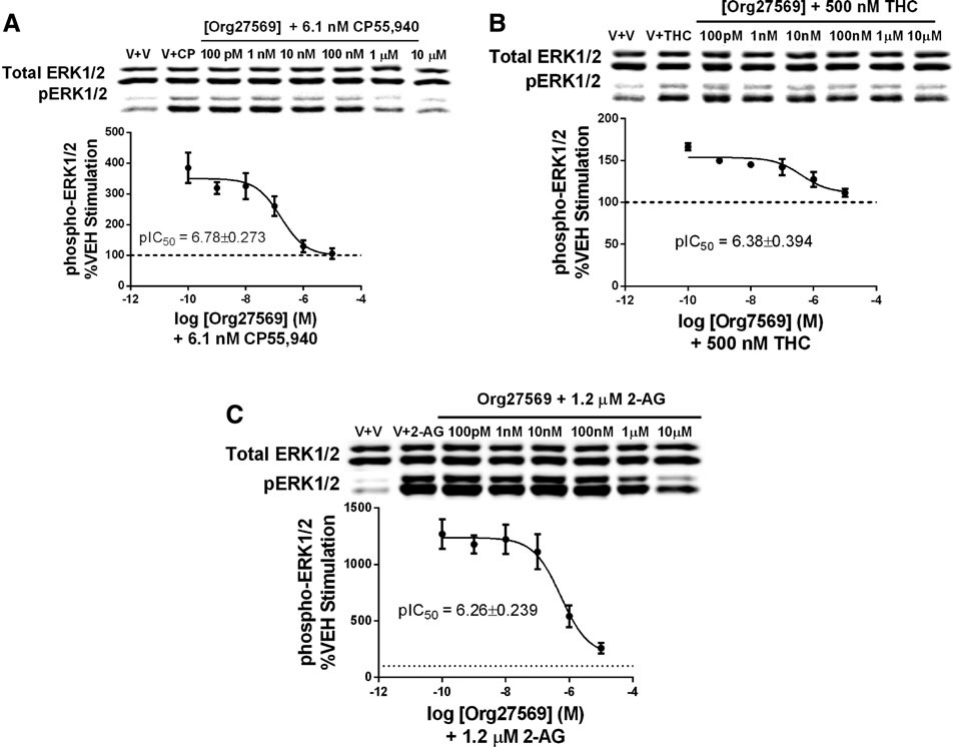
Org27569 antagonizes cannabinoid agonist-induced ERK activation. HEK293 cells stably transfected with the hCB_1_ receptor were exposed to specified agonists at their EC_80_ values and Org27569 for 5 min. Protein was separated by SDS-PAGE and western blots for phospho-ERK normalized to total ERK and curves were fitted using GraphPad Prism 5.0 to calculate IC_50_ values. **(A)** Org27569 concentration–response curve applied in combination with EC_80_ of CP55,940 (6.1 nM). **(B)** Org27569 concentration–response curve in the presence of EC_80_ THC (500 nM). **(C)** Org27569 concentration–response curve in the presence of EC_80_ 2-AG (1.2 μM). Data are the mean ± SEM of three independent experiments. CP, CP55,940; Org, Org27569; V, vehicle.

To determine the potency and extent of Org27569 ability to antagonize cannabinoid agonist-induced ERK activation, we first determined EC_80_ values for CP55,940 (EC_80_=6.1 nM), THC (EC_80_=500 nM), and 2-AG (EC_80_=1.2 μM). We then conducted full concentration–response curves for Org27569 in the presence of these agonists at their EC_80_ values (see Table 1 for summary of calculated IC_50_ values). Org27569 fully antagonized CP55,940-induced ERK activation (pIC_50_=6.78±0.273; Fig. 2A), while it did not completely attenuate THC-induced ERK activation (pIC_50_=6.38±0.394; Fig. 2B) or 2-AG-stimulated ERK activation (pIC_50_=6.26±0.238; Fig. 2C), with the bottom of the fit curves at 111.3%±7.72% and 196%±123.2%, respectively.

**Table 1.** Org27569 IC_50_ Values Against Agonists and Alone on ERK Phosphorylation

Probe agonist	IC_50_ (nM)	IC_50_ 95% confidence interval (nM)	pIC_50_±SE	pIC_50_ 95% confidence interval
CP55,940 (6.1 nM)^a^	165	43–629	6.783±0.273	7.36–6.2
THC (494 nM)^a^	417	60–2800	6.38±0.394	7.22–5.54
2-AG (1.2 μM)^a^	546	170–1760	6.26±0.239	6.77–5.76
None^b^	139	49–397	6.86±0.214	7.31–6.4

^a^ Cells treated for 5 min.

^b^ Cells treated for 20 min.

### Org27569 reduces basal ERK phosphorylation in both cytoplasmic and nuclear compartments via hCB_1_-G_i_ protein mechanism

**FIG. 3.**
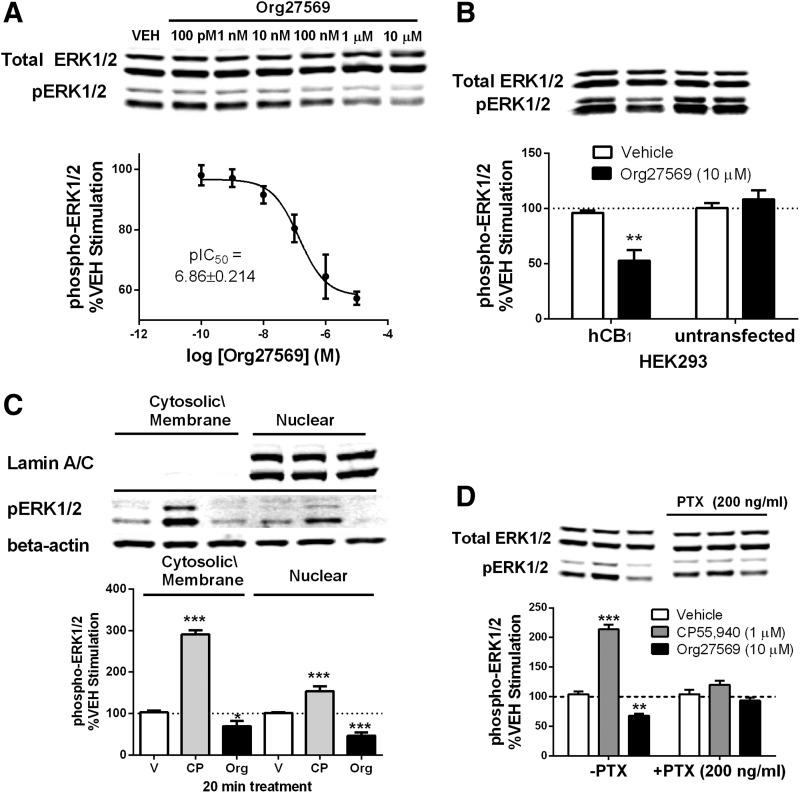
Org27569 (10.0 μM) reduces basal ERK phosphorylation in both cytoplasmic and nuclear compartments via hCB_1_-G_i_ protein mechanism following 20-min treatment. **(A)** Concentration–response curve for Org27569 alone. **(B)** Org27569 (10.0 μM) reduces basal phospho-ERK in hCB_1_-transfected but not hCB_1_-untransfected HEK293 cells. **(C)** Org27569 (10.0 μM) resulted in significant decreases in cytosolic and nuclear phospho-ERK. **(D)** Org27569 (10.0 μM) reduced basal ERK phosphorylation, which was prevented by overnight treatment with PTX. HEK293 cells stably transfected with the hCB_1_ receptor were exposed to Org27569 for 20 min. PTX (200 ng/mL) treatment was done overnight. Protein was separated by SDS-PAGE and western blots for phospho-ERK normalized to total ERK (except in fractions where it was normalized to beta-actin). Separate gels containing the same samples were run for immunoblotting of phospho-ERK/beta-actin and lamin A/C. Data were analyzed by one- or two-way ANOVA with Newman–Keuls and Bonferroni *post hoc* tests, respectively. Data are the mean ± SEM of at least three independent experiments. *p<0.05, **p<0.01, ***p<0.001. CP, CP55,940; Org, Org27569; PTX, pertussis toxin; V, vehicle.

We observed small but nonsignificant reductions in basal ERK phosphorylation with Org27569 (10 μM) alone at 5 min (Fig. 1A). Previously, the CB_1_ inverse agonist SR141716A was shown to reduce basal ERK phosphorylation following a 10-min incubation,^29,30^ and since we found that internalization was complete at 20 min (Fig. 1B), we next tested Org27569 using a 20-min incubation time. We observed that Org27569 significantly reduced basal ERK phosphorylation following a 20-min exposure (pIC_50_=6.86±0.21; Fig. 3A).

To determine receptor mediation, we tested Org27569 (10 μM) in hCB_1_-transfected and hCB_1_-untransfected HEK293 cells. We found that following a 20-min treatment, Org27569 reduced basal ERK phosphorylation in hCB_1_-transfected but not in hCB_1_-untransfected HEK293 cells (Fig. 3B) (main effect: Org27569 treatment [*F*(1,8)=6.765, *p*<0.05], cell type [*F*(1,8)=19.43, *p*<0.01] and significant interaction [*F*(1,8)=13.99, *p*<0.01]), suggesting that reductions in basal ERK phosphorylation occurred through the hCB_1_ receptor.

Org27569 has been reported to signal through beta-arrestin 1 to activate ERK.^23^ Beta-arrestin-mediated signaling through ERK has been suggested to occur through a large receptor–arrestin–ERK complex that cannot translocate into the nucleus, resulting in elevated cytosolic levels of phospho-ERK and decreased nuclear levels of phospho-ERK.^33^ A recent report showing that Org27569 alone did not activate ERK suggested that this may have been due to an inability to assess distinct pools of ERK (i.e., nuclear vs. cytosolic) at the subcellular level.^13^

To further delineate the effects of Org27569 at the subcellular level, fractionation experiments were conducted to determine if differences in ERK phosphorylation could be detected in these separate cellular compartments following Org27569 treatment. We observed that Org27569 (10 μM) significantly decreased cytosolic [*F*(2,9)=151.1, *p*<0.0001] and nuclear phospho-ERK levels [*F*(2,9)=41.27, *p*<0.0001] (Fig. 3C).

To further determine the mechanism of action of Org27569 effects, we examined the role of G_i/o_ proteins by preventing their activation with overnight treatment PTX (200 ng/mL). We observed that Org27569 produced a reduction in phospho-ERK levels in control cells but not in PTX-treated cells, suggesting a G_i/o_-dependent effect (main effect: treatment [*F*(2,18)=105.5, *p*<0.0001], PTX [*F*(1,18)=20.22, *p*<0.001], and significant interaction [*F*(2,18)=52.34]) (Fig. 3D).

## Discussion

Presently, Org27569 remains the most-studied and prototypical CB_1_ allosteric modulator, with newly synthesized compounds based on its structure being investigated for pharmacological properties. Org27569 and its structural analogs demonstrate positive binding cooperativity with the CB_1_/CB_2_ agonist CP55,940 and negative binding cooperativity with the selective CB_1_ antagonist/inverse agonist SR141716A. Functionally, Org27569 exhibits allosteric antagonism of CB_1_-mediated agonist-stimulated [^35^S]GTPγS binding and cAMP production.^13,14,20,21^ Furthermore, the Org27569 pharmacological profile is such that it decreases basal activity in both [^35^S]GTPγS^20,21,34^ and cAMP assays,^22^ suggesting that it is an inverse agonist of CB_1_-mediated G-protein signaling.

However, it has been reported that Org27569 can enhance or act as an allosteric agonist of ERK signaling via either G_i/o_^20^ or beta-arrestin 1.^23^ In contrast, others have reported that Org27569 acts as a CB_1_ allosteric antagonist of ERK activation.^13^ In addition to this, Org27569 has also been reported to antagonize agonist-induced suppression of forskolin-stimulated cAMP production^13,22^ and act as an inverse agonist of CB_1_-mediated inhibition of cAMP production, which was prevented by treatment with PTX.^22^

In this study, we report that Org27569 serves as an antagonist of CB_1_-mediated ERK signaling by the cannabinoid agonists CP55,940, THC, and 2-AG and also that it acts as an inverse agonist of basal ERK phosphorylation. These findings are mostly congruent with those recently reported by Khajehali et al.^13^ as we observed full antagonism of CP55,940-induced ERK activation, whereas inhibition of 2-AG-induced ERK activation was not complete.

While we observed that Org27569 antagonized THC-induced ERK activation, Khajehali et al.^13^ reported no effect. There are some differences between our study and theirs. First, they used Chinese hamster ovary cells, whereas we used HEK293 cells. Furthermore, Khajehali et al.^13^ assessed ERK activation with the AlphaScreen SureFire kit, whereas we measured phospho-ERK levels by western analysis, so it may be that westerns are more sensitive for detecting these changes. Our calculated IC_50_ values for Org27569 (Table 1) when tested against the EC_80_ values of orthosteric cannabinoid agonists were not significantly different from one another, suggesting that Org27569 does not exhibit biased antagonism of THC, 2-AG or CP55,940 in terms of ERK signaling.

In consideration of the established data indicating that Org27569 reduces basal [^35^S]GTPγS binding consistent with an inverse agonist, and that the selective CB_1_ antagonist/inverse agonist SR141716A reduces basal ERK activation in a PTX-sensitive manner,^30^ we further hypothesized that Org27569 would act as an inverse agonist of ERK signaling in our cell line. Following a 20-min incubation, Org27569 did indeed reduce basal ERK phosphorylation in hCB_1_ HEK293 cells but not in untransfected cells, suggesting that Org27569 acts as an hCB_1_ inverse agonist of ERK signaling. We observed that overnight treatment with PTX prevented the reduction in phospho-ERK, suggesting that the Org27569 inverse agonist effects result from reduced basal activity of the receptor coupling with G_i/o_.

Since HEK293 cells do not synthesize endocannabinoids and the cells were serum starved overnight before treatment, it is likely that the reduction in ERK phosphorylation following Org27569 treatment is due to inhibition of constitutive activity, consistent with an inverse agonist. ERK activation through CB_1_ by typical cannabinoid agonists has been shown to occur through G_i/o_ proteins,^35^ and therefore, these data are consistent with the known signaling properties of the CB_1_ receptor. Inverse agonism by Org27569 was not reported by Khajehali et al.^13^; this may be due to differences in CB_1_ expression between our cell lines since a higher expression level results in a greater basal activity making detection of inverse agonists easier.^36^

The lack of agonist effects of Org27569 alone on ERK signaling was previously suggested to be due to an inability to quantify pools of phospho-ERK, which may have a distinct subcellular localization.^13^ Indeed, ERK signaling by the angiotensin II type 1A (AT1a) receptor through a beta-arrestin mechanism was reported to result in an increase in cytosolic phosph-ERK with a concomitant reduction in nuclear phospho-ERK due to the inability of the receptor–arrestin–ERK complex to translocate into the nucleus.^33^

To test the hypothesis that Org27569 increases cytosolic phospho-ERK but decreases nuclear phospho-ERK, we performed subcellular fractionation of cells treated with Org27569 for 20 min to examine both nuclear and cytosolic phospho-ERK levels. We did not observe an increase in phospho-ERK levels following Org27569 in either the cytosolic or nuclear fractions, suggesting that Org27569 under these conditions does not serve as an agonist of ERK. We did observe decreases in phospho-ERK in the nuclear-enriched and cytosolic fractions, consistent with a reduction in basal ERK activation.

Previously, AT1a receptor signaling through beta-arrestin to activate ERK was observed following overexpression of beta-arrestins, which suggests that differences in levels of beta-arrestin expression between cell lines may explain the lack of observed biased agonist effects of Org27569 in our cell line. Activation of ERK by Org27569 was previously reported to be beta arrestin-1 dependent in HEK293 cells that were not overexpressing beta arrestin-1.^23^

The HEK293 cells used in our study were acquired from the American Type Culture Collection. These cells have been previously reported to express both beta arrestin-1 and beta arrestin-2,^37^ although this does not rule out the possibility of differences in expression level that may exist between our cell line and others. Nonetheless, these data are consistent with those one would expect from a CB_1_ receptor inverse agonist as the selective CB_1_ antagonist/inverse agonist SR141716A^38^ has been shown to serve as an inverse agonist at ERK in a PTX-sensitive manner.^30^ In addition, reported inverse agonist effects of Org27569 on cAMP production^22^ are also consistent with those of the CB_1_ inverse agonist SR141716A on adenylyl cyclase activity.^39,40^

We also tested whether Org27569 could prevent CP55,940-induced internalization of CB_1_ receptors. We found that CP55,940 resulted in an increase in puncta, consistent with receptor internalization, at each time point. Cotreatment with Org27569 prevented CP55,940-induced internalization at each time point. Org27569 alone did not significantly affect receptor internalization.

In summary, our data support previous studies that suggest Org27569 is an allosteric antagonist/inverse agonist of G-protein signaling.^13,14,20,22^ Consistent with the study by Khajehali et al.,^13^ we find that Org27569 acts as an antagonist of CB_1_-mediated ERK activation with the exception that we observed antagonism of THC-induced ERK1/2 phosphorylation, whereas Khajehali et al.^13^ did not. In contrast with studies by Ahn et al.^23^ and Baillie et al.,^20^ Org27569 did not serve as an agonist of ERK signaling as we observed no increases in phospho-ERK in either the cytosolic or nuclear-enriched subcellular fractions.

It is possible that levels of beta arrestin-1 in our cell line may be insufficient to allow biased coupling to this signaling pathway as reported by Ahn et al.^23^ Indeed, for the AT1a receptor, biased ERK signaling through beta arrestin was observed only following overexpression of beta arrestin1 and beta-arrestin 2.^33^ Thus, differences in beta-arrestin expression between cell lines may account for the differences observed in Org27569 effects when administered alone. In addition, we report that Org27569 exhibits inverse agonism of ERK signaling and that this is through the hCB_1_ receptor via G_i/o_ proteins. To our knowledge, this is the first report of inverse agonism of ERK signaling by Org27569; this adds to the reported inverse agonism of Org27569 on [^35^S]GTPγS binding^14,20,21^ and cAMP.^22^

## Abbreviations Used

AT1a: angiotensin II type 1A
CB_1_: cannabinoid type-1 receptors
CB_2_: cannabinoid type-2 receptors
DMEM: Dulbecco's modified Eagle's medium
ERK: extracellular signal-regulated kinase
FBS: fetal bovine serum
GFP: green fluorescent protein
GPCR: G-protein-coupled receptors
hCB_1_: human CB_1_
PBS: phosphate*-*buffered saline
PDL: poly-d-lysine
PTX: pertussis toxin
